# Proton-coupled monocarboxylate transporters in cancer: From metabolic crosstalk, immunosuppression and anti-apoptosis to clinical applications

**DOI:** 10.3389/fcell.2022.1069555

**Published:** 2022-11-23

**Authors:** Qixin Duan, Shuang Zhang, Yang Wang, Dongming Lu, Yingming Sun, Yongyang Wu

**Affiliations:** ^1^ Department of Urology, Affiliated Sanming First Hospital of Fujian Medical University, Sanming, Fujian, China; ^2^ Department of Urology, Nanyang Central Hospital, Nanyang, China; ^3^ Department of Nursing, Nanyang Central Hospital, Nanyang, China; ^4^ Department of Medical and Radiation Oncology, Affiliated Sanming First Hospital of Fujian Medical University, Sanming, Fujian, China

**Keywords:** monocarboxylate transporters, lactate, tumor metabolism, metastasis, angiogenesis, tumor microenvironment, autophagy, MCT inhibitors

## Abstract

The Warburg effect is known as the hyperactive glycolysis that provides the energy needed for rapid growth and proliferation in most tumor cells even under the condition of sufficient oxygen. This metabolic pattern can lead to a large accumulation of lactic acid and intracellular acidification, which can affect the growth of tumor cells and lead to cell death. Proton-coupled monocarboxylate transporters (MCTs) belong to the SLC16A gene family, which consists of 14 members. MCT1-4 promotes the passive transport of monocarboxylate (e.g., lactate, pyruvate, and ketone bodies) and proton transport across membranes. MCT1-4-mediated lactate shuttling between glycolytic tumor cells or cancer-associated fibroblasts and oxidative tumor cells plays an important role in the metabolic reprogramming of energy, lipids, and amino acids and maintains the survival of tumor cells. In addition, MCT-mediated lactate signaling can promote tumor angiogenesis, immune suppression and multidrug resistance, migration and metastasis, and ferroptosis resistance and autophagy, which is conducive to the development of tumor cells and avoid death. Although there are certain challenges, the study of targeted drugs against these transporters shows great promise and may form new anticancer treatment options.

## Introduction

Monocarboxylic acids represented by lactate, pyruvate, and ketone bodies are essential metabolites in most mammalian cells, and their dynamic absorption and redistribution are carried out through monocarboxylate transporters (Felmlee et al., 2020). There are two types of monocarboxylate transporters in the body: proton-coupled monocarboxylate transporters (MCTs) and sodium-coupled monocarboxylic monocarboxylate transporters (SMCTs). MCTs belong to the solute carrier family 16 (SLC16) or MFS superfamily, which includes 12 transmembranes (TMs) helices with intracellular N- and C-termini, a large cytosolic loops between TM6 and TM7, and two highly conserved sequences in TM1 and TM5 (Halestrap, 2012; Halestrap, 2013). Among the 14 members of the family, MCT1/SLC16A1, MCT2/SLC16A7, MCT3/SLC16A8, and MCT4/SLC16A3 convey monocarboxylate ions together with protons. These passive transporters are primarily localized at the plasma membrane, where they can operate bidirectionally depending on the concentration gradient of their substrates (Payen et al., 2020). SLC16A2 encodes the high-affinity thyroid hormone transporter (MCT8) and the SLC16A10 aromatic amino acid transporter (TAT1), while the substrates and roles of the remaining eight members are unknown (Adijanto and Philp, 2012).

Since the members of the MCTs family are themselves unglycosylated, MCT1-4 requires binding the glycosylated accessory proteins (Basigin/CD147 or embigin) to maintain their activity (Payen et al., 2020). Basigin (also known as CD147 or EMMPRIN) is a molecular chaperone that assists in MCT1, 3, and 4 localization, and gp70/embigin is a molecular chaperone in MCT2. Interactions of MCT1 with gp70 and MCT2 with CD147 have also been observed, which it maybe species dependent (Ovens et al., 2010a). The stability of MCTs and their associated chaperone proteins is interdependent, as silencing of one usually reduces the expression of the other (Marchiq et al., 2015).

Different MCTs isoforms have different affinities for the same substrate (Payen et al., 2020). MCT1 has a high affinity for lactate (3–6 mM), and the direction of lactate transport driven by MCT1 depends on the gradient of lactate and proton across the membrane and metabolic state (Van Hée et al., 2017; Halestrap and Wilson, 2012). The main physiological role of MCT1 is to transport L-lactate into cells for gluconeogenesis or oxidative phosphorylation, but MCT1 is a major lactate exporter in some normal cells under certain circumstances, including white skeletal muscle fibers, red blood cells, astrocytes, oligodendrocytes, hypoxic cells, and immune cells such as activated T lymphocytes (Halestrap, 2012). In solid tumors, the oxygen utilization and distribution lead to tumor symbiosis, where lactate secreted by tumor cells in the hypoxic zone (its concentration can be as high as 40 mM (Dhup et al., 2012) is more absorbed by perivascular tumor cells through MCT1, promoting tumor growth and proliferation (Potter et al., 2016). It has also been reported that MCT1 can promote lactate export from cancer cells (Morais-Santos et al., 2015). Both MCT2 and MCT3 have been poorly studied. MCT2 is expressed in brain, liver, and renal tubules, whereas MCT3 is in the choroid plexus and retina. MCT4 has a low affinity for lactate (25–30 mM) and does not intake serum lactate (usually <2 mM). MCT4 gives cells the ability to export lactate in a high-lactate micro-environment, which it has physiological relevance to pyruvate (Contreras-Baeza et al., 2019. Current studies have shown that MCT1, MCT2 or MCT4 are widely expressed in tumor cell lines and patients with various tumor types, and the expression of MCT subtypes or CD147 is significantly increased in most tumor cells compared with the adjacent normal epithelium (Choi et al., 2014; Pinheiro et al., 2014; De Oliveira et al., 2012). (Table 1). However, compared with normal tissue, the expression of MCT2 and MCT4 in prostate tumor cells were significantly increased, while the expression of MCT1 and CD147 significantly decreased (Pertega-Gomes et al., 2011). Although the expression of MCT4 gradually increased from non-neoplastic tissues to HCC and to metastasis, the overall expression of MCT2 gradually decreased (Alves et al., 2014). In addition, high expression of MCT1, MCT2, MCT4 or CD147 is associated with advanced tumor nodal metastasis (TNM) stage and tumor prognosis, and is a marker of poor tumor prognosis (Pertega-Gomes et al., 2011; Pinheiro et al., 2016; Payen et al., 2020). (Table 2). However, MCT2 expression suggests a better prognosis in hepatocellular carcinoma (Alves et al., 2014). MCTs play an important role in the growth, metabolism, proliferation, metastasis, and immune tolerance oftumor cells (Doherty and Cleveland, 2013; Payen et al., 2020), and more effort has been devoted to the development of MCT inhibitors as potential anti-cancer agents.

**TABLE 1:** Changes of MCTs expression in human cancers comparing with normal tissues.

Cancer type	MCT1	MCT2	MCT4	CD147	Ref
urothelial carcinoma of the bladder	high		high	high	Choi et al. (2014)
clear cell renal cell carcinoma	high		high	high	Kim et al. (2018)
prostate cancer	high	high	high	low	Pertega-Gomes et al. (2011)
non-small cell lung cancer	high		high		Tong et al. (2021)
T-cell non-Hodgkin lymphoma	high		high		Zhao et al. (2022)
head and neck cancer	uncertain		high		Curry et al. (2013)
gastric cancer	high		uncertain	high	Eskuri et al. (2021)
cervical carcinoma	high		high	high	Pinheiro et al. (2009)
pancreatic ductal adenocarcinoma	high		high		Kong et al. (2016)
melanomas	uncertain		high		Pinheiro et al. (2016)
endometrial cancer	high		high	high	Latif et al. (2017)
glioblastomas	high		high	high	Miranda-Gonçalves et al. (2013)
soft tissue sarcomas	high	high	high	high	Pinheiro et al. (2014)
gastrointestinal stromal tumors	high	high	high	high	de Oliveira et al. (2012)
breast cancer	high		high		Yuan et al. (2021)
basal-like breast cancer	high		high		Pinheiro et al. (2010)
testicular germ cell tumors	high		high	high	Silva et al. (2018)
cutaneous squamous cell carcinoma	uncertain		high	high	Sweeny et al. (2012)
Colorectal carcinomas	high	high	high		Pinheiro et al. (2008), Alves et al. (2014)
Hepatic carcinoma cells		low	high		Alves et al. (2014)

**TABLE 2:** Prognostic value of MCTs expression in human cancers.

Cancer type	MCT1	MCT2	MCT4	CD147	Ref.
urothelial carcinoma of the bladder	bad		uncertain	bad	Choi et al. (2014)
clear cell renal cell carcinoma	bad		uncertain	bad	Kim et al. (2018)
prostate cancer	bad	uncertain	bad	bad	Pertega-Gomes et al. (2011)
non-small cell lung cancer			bad		Tong et al. (2021)
T-cell non-Hodgkin lymphoma	bad		uncertain		Zhao et al. (2022)
head and neck cancer			bad		Curry et al. (2013)
gastric cancer	bad			bad	Eskuri et al. (2021)
cervical carcinoma	bad			bad	Pinheiro et al. (2009)
pancreatic ductal adenocarcinoma	bad				Kong et al. (2016)
melanomas	bad		bad		Pinheiro et al. (2016)
endometrial cancer	bad				Latif et al. (2017)
soft tissue sarcomas	bad				Pinheiro et al. (2014)
gastrointestinal stromal tumors	bad			bad	de Oliveira et al. (2012)
breast cancer			bad		Yuan et al. (2021)
basal-like breast cancer	bad		bad		Pinheiro et al. (2010)
testicular germ cell tumors			bad		Silva et al. (2018)
cutaneous squamous cell carcinoma				bad	Sweeny et al. (2012)
hepatic carcinoma cells		good	bad		Alves et al. (2014)

## MCT and tumor metabolism

### MCT and energy metabolism

Generally speaking, most cells in the body produce adenosine triphosphate (ATP) by oxidative phosphorylation (OXPHOS) in mitochondria and complete oxidation of glucose to CO_2_, producing 32–38 mol of ATP per mole of glucose (Puri and Juvale, 2020). However, some cells exposed to hypoxia and proliferating cells or tumor cells show a transition from oxidative phosphorylation to anaerobic glycolysis and aerobic glycolysis, respectively, which preferentially convert glucose to lactate, producing only 2 ATPs per glucose molecule. (Vander Heiden et al., 2009). The rate of glucose metabolism through aerobic glycolysis is faster relative to OXPHOS, which is 10 to 100 times faster than the complete oxidation of glucose in mitochondria (Liberti and Locasale, 2016). Most tumor cells still provide energy by hyperactive glycolysis in the presence of sufficient oxygen, which promotes their rapid growth and proliferation, namely the Warburg effect (Vaupel et al., 2019).

Solid tumors are composed of two different types of cells. Normoxic or oxidative tumor cells are close to blood vessels and have high oxygen content around the cells. On the contrary, hypoxic cells or glycolytic tumor cells are far away from blood vessels and low oxygen content (Rockwell et al., 2009). Glycolytic tumor cells enhance the expression of glucose transporter 1 (GLUT1) to overcome ATP deficiency and accelerate glucose uptake. HIF- 1 facilitates the conversion of glucose to pyruvate and subsequently to lactate in the presence of lactate dehydrogenase 5 (LDH5) promoting NAD regeneration, which is required to maintain high glycolytic flux (Nagao et al., 2019). Lactate and hypoxia-stimulated HIF- 1α or WNT/β-catenin signaling prevent pyruvate from entering the TCA cycle and converting to acetyl-CoA by upregulating pyruvate dehydrogenase kinase 1 (PDK1) and then inhibiting pyruvate dehydrogenase complex (PDH), and drive tumor cells to obtain energy by leading to aerobic glycolysis (Kim et al., 2006; Vallee et al., 2017). In hepatocellular carcinoma, glycolysis is four times more potent than oxidative phosphorylation (Beyoğlu et al., 2013). Finally, MCT4 removes lactate from glycolytic tumor cells to avoid intracellular acidification. The low expression level of HIF- 1 and allosteric feedback inhibition of lactate in oxidized tumor cells result in low glycolysis efficiency (Leite et al., 2011). To meet energy requirements, oxidized tumor cells use MCT1 to take up lactate, which is oxidized by LDH- 1 to pyruvate, while NAD+ is reduced to NADH.

In many cases, mitochondrial oxidative phosphorylation (OXPHOS) still contributes to ATP production by tumor cells (Fu et al., 2017). Oxidized tumor cells utilize the breakdown products of glycolysis, including lactate, pyruvate, and NADH (which fuels the mitochondrial electron transport chain (ETC) *via* the apple-aspartic acid shuttle) to generate ATP through the tricarboxylic acid (TCA) cycle and mitochondrial OXPHOS (Ganapathy-Kanniappan and Geschwind, 2013; Van Hee et al., 2015). For oxidized tumor cells, oxidative lactate metabolism is more advantageous than aerobic glycolysis, and each molecule consumed by lactate generates 7.5 times more ATP produced (Payen et al., 2020).

**FIGURE 1:**
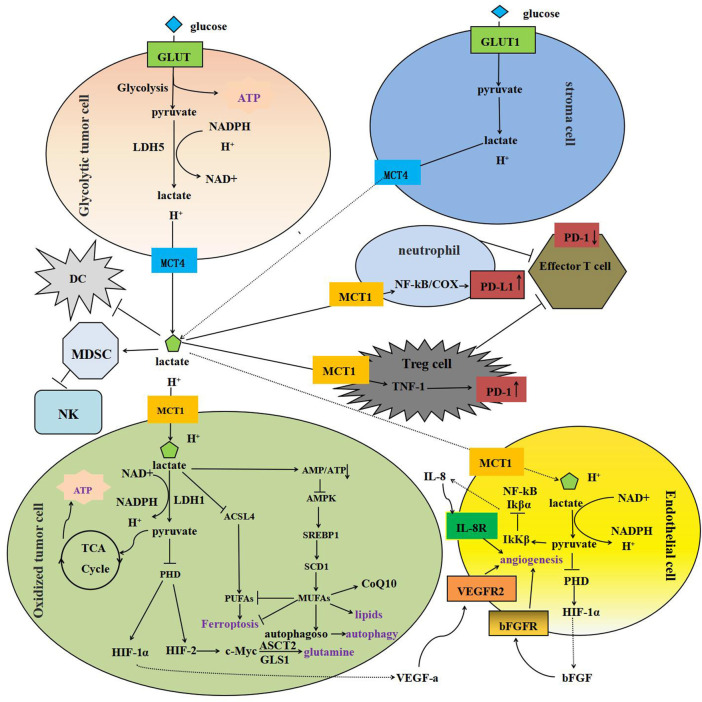
Model depicting MCT-mediated lactate transport in cancer: metabolic crosstalk, immunosuppression, tumor angiogenesis and anti-apoptosis. **(A)** MCT1-4-mediated lactate shuttling between glycolytic tumor cells and oxidized tumor cells Firstly, glycolytic tumor cells and stromal cells in the hypoxic region produce lactate and hydrogen ions by glycolysis, which are excreted into the tumor microenvironment by MCT4, and then taken up by oxidized tumor cells expressing MCT1. It is reduced to pyruvate and NANPH by LDH and enters the TCA. In addition, MCT1-mediated lactate intake can lead to the disruption of AMP/ATP balance and the inactivation of AMPK, and up-regulate the expression of SCD1 through the AMPK-SREBP1 pathway to promote the biosynthesis of MUFAs. MUFAs are substrates for the synthesis of various lipids. Finally, lactate ingestion *via* MCT1 stimulates glutamine uptake and catabolism by inhibiting PHD, stabilizing HIF-2α, transactivating c-Myc, and upregulating ASCT2 and GLS1 expression. **(B)** MCT-mediated lactate transport inhibits immune cells MCT-mediated lactate transport affects the maturation of DCS by reducing the efficiency of NF-κB binding to DNA, promotes the differentiation of peripheral blood mononuclear cells in MDSCs, and reduces the activity of NK cells. MCT-mediated lactate intake promotes the translocation of activated TNF-1 to the nucleus and up-regulates the expression of PD-1 in Tregs, but inhibits in effector T cells. PD-1 blockade activates PD-1-expressing Treg cells and inhibits effector T cells. In addition, lactate enters neutrophils through MCT1 and induces the expression of PD-L1 through NF-κB/COX-2 pathway, which reduces the cytotoxicity of T cells. **(C)** MCT-mediated lactate transport promotes tumor angiogenesis In oxidized tumor cells and endothelial cells, lactate is taken up *via* MCT1 and oxidized to pyruvate, and then inhibits PHD and catalyzes the hydroxylation of HIF-1α, which stimulates the transcription of vascular VEGF-a, VEGFR2 and bFGF. In addition, pyruvate can regulate the expression and activity of IκKβ, leading to phosphorylation and subsequent proteasome degradation of IκBα, nuclear translocation of NF-κB and transcription of the proangiogenic factor IL-8. VEGF-a, VEGFR2, bFGF and IL-8 are all pro-angiogenic factors with good characteristics.

The lactate shuttle mediated by MCT1 and MCT4 can connect cancer cells with glycolysis in Glycolytic tumor cells and mitochondrial oxidation in oxidized tumor cells as the main production mode, and make them form cooperative metabolism, promoting the occurrence and development of tumors (Wang et al., 2022) (Figure 1).

The metabolic synergy of tumor tissue also involves the glycolysis of stromal cells, such as cancer-associated fibroblasts (CAFs), and the lactate released by MCT4 provides the raw material for the oxidative metabolism of cancer cells (Wilde et al., 2017). This symbiotic relationship between cancer cells and stromal cells is called the “reverse Warburg effect” (Benny et al., 2020).

### MCT and lipid metabolism

Lactate shuttle mediated by MCT is not merely a metabolic waste product but a nutrient with multiple regulatory roles in the tumor microenvironment, and it is particularly relevant to lipid biosynthesis as a major carbon source (Faubert et al., 2017; Chen et al., 2016).

Increasing lactate concentration in the tumor microenvironment could facilitate MCT1-mediated lactate uptake on the cytoplasmic membrane, which would promote ATP production and decrease the AMP: ATP ratio in the intracellular compartment (Yan et al., 2021). The lactate-induced disruption of AMP: ATP balance would further deactivate the energy sensor AMP-activated protein kinase (AMPK), leading to upregulating the expression of the sterol regulatory element-binding protein 1 (SREBP1) and increasing the stearoyl-coenzyme A (CoA) desaturase- 1 (SCD1) expression through the AMPK- SREBP1 pathway (Li et al., 2022). It has also been reported that SCD1-catalyzed monounsaturated fatty acids (MUFAs) may replace polyunsaturated fatty acids (PUFAs) in the lipid membrane and reduce the accumulation of cytotoxic lipid ROS (Zhao et al., 2020). SCD1 promotes the biosynthesis of MUFAs (i.e., palmitoleate and oleate) from their SFA precursors (i.e., palmitate and stearate). MUFAs are substrates for the synthesis of various lipids, including phospholipids (PLs), diacylglycerol (DAGs), triacylglycerol (TAGs), and cholesteryl esters (CEs), which are essential components of biological membranes as well as cellular energy sources and signaling molecules (Ascenzi et al., 2021) (Figure 1). It is confirmed that MCT1 inhibition with AZD3965 inhibits phospholipid biosynthesis and decreases choline-phospholipid metabolism *in vivo* tumors (Beloueche-Babari et al., 2020).

Otherwise, MCT1 knockdown has significantly reduced the production of phospholipids containing MUFAs and down-regulated the expression of Coenzyme Q10 (CoQ10), an important enzyme in lipid metabolism and the downstream product of SCD1, while the pro-ferroptosis phosphatidylethanolamine (PE) has increased in varying degrees (Tesfay et al., 2019).

In addition, MCT1 is widely distributed in various tissues and cells. In the intestinal epithelium, its localization at both apical and basolateral membranes has been considered to promote the absorption of short-chain fatty acids (SCFAs) produced by the anaerobic intestinal bacterial fermentation of dietary fiber and resistant starch (Van Rymenant et al., 2017; Ziętek et al., 2021). SCFAs, especially acetate, propionate, and butyrate, not only provide substrates for lipid synthesis but also act as regulators to regulate lipid metabolism (He et al., 2020). MCT1 as an acetate transporter increases acetate uptake (Jeon et al., 2018). Exogenous acetate is converted to acetyl-CoA by cytosolic acetyl- CoA synthetase for lipid synthesis in acetate-dependent tumors (Lyssiotis and Cantley, 2014). Moreover, the expression of acyl-CoA synthetase 2 and fatty acid synthase, the key enzymes of controlling lipid synthesis, are elevated in the presence of acetate in HepG2 cells (Jeon et al., 2018).

### MCT and amino acid metabolism

Rapidly proliferating cancer cells usually show an increased dependence on amino acid metabolism (Contreras-Baeza et al., 2019). Tumor cells are involved in various crucial biochemical functions inthe brain and other tissues, such as protein synthesis and energy production, through the uptake of glutamine and the branched-chain amino acids (BCAAs) valine, leucine, and isoleucine (Jain et al., 2012). MCT1 but not MCT4 mediates the effusion of BCAAs and branched-chain ketoacids (BCKAs)-branched-chain amino acid catabolite, which may play a role in tumor immunosuppression (Silva et al., 2017).

Lactate transport across the cell membrane is mainly promoted by passive transporters of the MCT family (Halestrap and Wilson, 2012). Lactate and hydrogen ions produced by glycolytic tumor cells in the hypoxic region are excreted into the tumor microenvironment through MCT4 and then taken up by peripheral oxidized tumor cells expressing MCT1. Due to the inward gradient of lactate and protons, oxidized cancer cells are able to import lactate for signaling, whereas glycolytic cancer cells that produce and export lactate do not take up lactate and show intracellular signaling in response to exogenous lactate (De Saedeleer et al., 2012).

Lactate intake through MCT1 acts as a paracrine signaling agent that generates a pseudohypoxic response in oxidized cancer cells where it not only leads to HIF- 1α but also HIF-2α stabilization and HIF-2 activation after oxidation to pyruvate and pyruvate-mediated inhibition of proguanidin hydroxylase (PHD) (Lu et al., 2005; Perez-Escuredo et al., 2016a). It was reported that HIF-2α mediates c-Myc transactivation, which promotes glutamine uptake and metabolism through enhancing expression of the inward glutamine transporter ASCT2 and the glutamine-metabolizing enzyme glutaminase 1 (GLS1) (Perez-Escuredo et al., 2016b). (Figure 1). Furthemore, lactate-induced glutamine metabolism has been shown to activate mTOR, a key nutrient sensor and master regulator of cell growth, which stimulates protein synthesis (Allen et al., 2016).

## MCT, tumor microenvironment and immunity

### MCT and tumor microenvironment

The tumor microenvironment (TME) is the internal environment for the generation and growth of tumor cells. It includes not only tumor cells themselves, but also cells and interstitial cells closely related to tumor cells (Arneth, 2019). Poor vascular differentiation in the TME results in inefficient oxygen and nutrient delivery and metabolic waste removal, leading to nutrient limitation, low pH, hypoxia, and metabolic accumulation (Bader et al., 2020).

Rapidly proliferating tumor cells compete for relatively scarce nutrients with immune cells for anti-tumor defense, aking tumor cells themselves create an anti-immune metabolic microenvironment. Lactate produced by tumor cells is excreted by MCT and plays an immunosuppressive role in the tumor microenvironment. Lactate accumulation in the TME inhibits effector T cell function by decreasing T cell proliferation and IFN-γ production, and activates G protein-coupled receptor 81 (GPR81) on immune cells and endothelial cells, promoting angiogenesis and immune escape (Brand et al., 2016; Brown and Ganapathy, 2019). Lactate affects the maturation of dendritic cells (DCs) by reducing the efficiency of NF-κB binding to DNA and significantly promotes the differentiation of monocyte-derived DCs, which reduces glucose consumption, upregulates mitochondrial respiratory genes, and inhibits mTORC1 activity (Puig-Kroger et al., 2003). Lactate can also promote the differentiation of peripheral blood mononuclear cells in myeloid-derived suppressor cells (MDSCs), reducing the activity of NK cells, and promoting immunosuppression (Husain et al., 2013). Molecularly, Lactate can inhibit the expression of NFAT in T cells and NK cells (Puig-Kroger et al., 2003).

Activated cytotoxic T lymphocytes (CTLs) depend on glycolysis and export lactate through MCT1. However, MCT is a passive transporter whose activity is driven by the concentration of lactate and protons across the cell membrane. The accumulation of lactate in the TME prevents the efflux of lactate from these cells and impairs glycolytic activity, proliferation, and function of cytotoxic T lymphocytes and activated monocytes, thereby promoting immune resistance (Dietl et al., 2010; Fischer et al., 2007). Given the importance of MCT1 for CTL proliferation in immune responses, MCT1 inhibitors were initially developed as immunosuppressants to inhibit tissue graft rejection. Extracellular lactic acid inhibits HDACs activity, leading to histone hyperacetylation, reduced chromatin compactness, and altered gene expression, which plays an important role in promoting DNA repair and promoting chemotherapy resistance in cancer cells. Both GPR81 silencing and MCT1 inhibition can interfere with this process (Wagner et al., 2015) (Figure 1).

### MCT and immune checkpoints

MCT also has an important effect on the expression of immune checkpoints such as PD- 1 on the surface of immune cells. Rapidly proliferating tumor cells enhance the expression of glucose transporter 1 (GLUT1) and accelerate glucose absorption, while regulatory T cells (Tregs) are forced to ingress lactate through MCT1, which not only maintains its growth and metabolism but also promotes the translocation of activated T nuclear factor 1 (TNF- 1) to the nucleus. Thus, the expression of PD- 1 is enhanced in Tregs but inhibited in effector T cells (Kumagai et al., 2022). PD- 1 blockade activates PD- 1-expressing Treg cells and inhibits effector T cells, including CD8^+^ T cells, which play a crucial role in killing cancer cells in the host, ultimately leading to treatment failure (Wherry and Kurachi, 2015). Combined treatment with MCT antibody and anti-PD- 1 can effectively inhibit tumor growth (Zhou et al., 2022).

Neutrophils are the most abundant inflammatory cells in peripheral blood. Under the stimulation of chemokines, neutrophils rapidly migrate to TME to play a role. On the one hand, reactive oxygen species (ROS), hydrogen peroxide, and tumor necrosis factor (TNF) related apoptosis-inducing ligands are released to attack tumor cells. On the other hand, neutrophils release inflammatory factors that stimulate angiogenesis, regulate tumor immunity, and promote tumor development and invasion. MCT1 and MCT4 are the major lactate receptors on neutrophils, not GPR81 (Khatib-Massalha et al., 2020). Lactate enters neutrophils through MCT1 and induces the expression of PD-L1 through NF-κB/COX-2 pathway. At the same time, lactate-H + can prolong the life span of neutrophils and partially promote the expression of PD-L1 and reduce T cell cytotoxicity (Deng et al., 2021).

### MCT and multidrug resistance

There are several mechanisms identified for the development of MDR in cancer, such as loss of drug targets, increased DNA repair mechanisms, decreased uptake of the drug, and increased drug efflux due to overexpression of ATP binding cassette (ABC) transporters (Gillet and Gottesman, 2010). Apart fromthese, one of the important MDR mechanisms commonly found in solid tumors is the altered metabolism, mainly at the level of cellular glycolytic (Alfarouk et al., 2014).

Generally, solid tumors consist of two heterogeneous cell types, in which normoxic cells are close to the blood vessels and highly oxygenated, and conversely, hypoxic cells are far from the blood vessels and deficient in oxygen (Rockwell et al., 2009). Hypoxic cancer cells with enhanced expression of GLUT1 overcome ATP deficiency by accelerating glucose uptake and thereby increasing the overall ATP production. HIF- 1 also facilitates the conversion of glucose to pyruvate, later converted to lactate by a well-expressed enzyme lactate dehydrogenase (LDH) (Nagao et al., 2019). To avoid cells death caused by lactate-mediated intracellular acidification, lactate is excreted from hypoxic cells by MCT4, and subsequently, these exported lactate molecules are taken up by well-oxygenated cells *via* MCT1, utilizing these input lactate as an alternative fuel for energy production (Park et al., 2018). Aerobic tumor cells contain low levels of HIF- 1, resulting in inefficient glycolysis. Therefore, to meet the energy demand, these cells utilize lactate produced by hypoxic cells and oxidize it to pyruvate *via* LDH- 1 while reducing NAD + to NADH. The generated pyruvate and NADH enter the TCA cycle and eventually undergo OXPHOS to generate ATP.

As less oxygen is available in hypoxic regions of the tumor, they develop an alternative method, a high rate of glycolysis, to produce energy. Upon the occurrence of a hypoxic TME, instead of weakening, tumor cells adapt to hypoxic conditions, which leads to more aggressive proliferation and the development of a therapy-resistant phenotype (Rohwer and Cramer, 2011).

## MCT and metastasis

In the absence of other nutrients, glucose deprivation drives tumor cells to migrate towards serum and glucose, in which MCTI, MCT4, and their chaperone proteins play an important role. It has been shown that MCT1 activates the transcription factor NF-κB, which is a protein complex and gene regulator that controls cell proliferation and cell survival and promotes tumor cell metastasis (Zhao et al., 2014). For renal clear cell carcinoma, MCT1 can drive lipid desaturation through the AMPK- SREBP1-SCD1 pathway to regulate the NF-κB signaling pathway, and promote tumor cell growth and migration (Yang et al., 2018). Silencing MCT1 can inhibit the NF-κB signaling pathway and the migration and metastasis of breast cancer cells while restoring MCT1 expression restore the NF-κB activation-dependent migration of cancer cells (Vegran et al., 2011). MCT1 inhibitors such as AR-C155858 and AZD3965 effectively inhibit the activity of transporters and reduce lactate transport, but they do not inhibit the migration and invasion of cancer cells. This indicates that MCT1 activates the transcription factor NF-κB to promote cancer cell migration independently of its transporter activity (Payen et al., 2017).

Glucose deprivation, which occurs in tumors, reduces essential fuel for mitochondria and promotes oxidative stress (Ferretti et al., 2012). The survival of tumor cells depends on autophagy and the ability to resist excessive ROS production, which induces the overexpression of MCT1 and CD147 after translation in a MTROs-dependent manner (De Saedeleer et al., 2014). CD147 can trigger the migration and metastasis of cancer cells by activating matrix metalloproteinases (MMPs) (Kumar et al., 2019). Thereby, it is providing a functional complex known to be involved in the transport of monocarboxylic acids (through MCT1) and the activation of MMPs (through CD147). Upon glucose starvation, the complex promotes tumor cell migration toward serum and glucose (De Saedeleer et al., 2014).

In humans, high MCT1 and MCT4 expression are usually associated with poor prognosis (Payen et al., 2020). MCT4 knockdown can result in abnormal transport and accumulation of CD147 in lysosomes, increase focal adhesion size, upregulate epithelial markers and downregulate mesenchymal markers (Gallagher et al., 2007; Zhu et al., 2014). Loss of MCT4 activity can locally alter transmembrane pH gradient and integrin signaling pathway and cell adhesion, leading to the migration and invasion of cancer cells (Gallagher et al., 2007).

Most previous studies have relied on silencing or inhibition of MCT1/MCT4 expression or activity in highly metastatic cell lines to investigate the relationship between MCTs and metastasis. However, silencing can be transient or incomplete, and inhibition can also have off-target effects. Whether cancer cells are dependent on both MCT1 and MCT4 remains uncertain. Some studies have used the non-tumorigenic mouse NCTC clone 929 (L929) cell line to express endogenous MCT1 and MCT4 and transfect MCT1 and MCT4, respectively. The results showed that overexpression of MCT4, but not MCT1, promoted the migration and invasion of L929 cells (Li et al., 2021). MCT4 can also promote the proliferation, migration, invasion, and epithelial-mesenchymal transition (EMT) of liver cancer cells by upregulating the transport protein particle complex subunit 5 (TRAPPC5) gene (Niu et al., 2022). Furthermore, MCT4 overexpression enhances cell migration and invasiveness by reorganizing the actin cytoskeleton (Reuss et al., 2021).

## MCT and tumor angiogenesis

In oxidized cancer cells and endothelial cells, lactate is taken up *via* MCT1 and oxidized to pyruvate, which competes with α-ketoglutarate to inhibit proline hydroxylases (PHDs) and catalyze the hydroxylation of HIF- 1α at two proline residues. This activates HIF- 1 and stimulates the transcription of vascular endothelial growth factor (VEGF-A), VEGF receptor 2 (VEGFR2), and basic fibroblast growth factor (bFGF) (Sonveaux et al., 2012; Payen et al., 2015). In addition, compared with the typical HIF1-dependent angiogenic factor VEGF produced by tumor cells or tumor-associated macrophages, endothelial cells also have an autocrine pathway for angiogenesis. Inhibition of PHD by lactate-derived pyruvate not only stabilizes HIF- 1α, but also regulates the expression and activity of an inhibitor of κB-kinase β (IκKβ), leading to phosphorylation of inhibitor of κBα (IκBα) and subsequent proteasomal degradation, and nuclear translocation of NF-κB and transcription of the proangiogenic factor interleukin-8 (IL-8) (Vegran et al., 2011). VEGF-A, VEGFR2, bFGF, and IL-8 are all pro-angiogenic factors with favorable properties, which activate their receptors respectively, stimulate endothelial cell proliferation and migration, and promote the generation of new blood vessels. Targeting MCT1 in endothelial cells can inhibit lactate-induced HIF- 1 activation and tumor angiogenesis (Sonveaux et al., 2012).

Lactate released from MCT4 by glycolytic tumor cells is absorbed by MCT1, thereby supporting pro-angiogenic signaling through HIF- 1α and autocrine NF-κB/IL-8 pathways (Reuss et al., 2021). At the same time, lactate is transported from tumor cells to the extracellular matrix, which increases the acidity of the extracellular environment and maintains the acidic tumor microenvironment. This transition leads to the activation of many cytokines by matrix metalloproteinases or other proteinases, such as IL-8, VEGF, and angiopoietin 2 (ANGPT2), which promote tumor neovascularization (Pouyssegur et al., 2006; Vegran et al., 2011; Xiao et al., 2020). Co-expression of MCT subtypes with VEGF family members has been demonstrated, such as the association between MCT1 and VEGF-C and between MCT4 and VEGF-A and VEGFR-3 (Pinheiro et al., 2015). In addition, MCT4 and VEGF can be regulated by HIF- 1α. Hypoxic environment is first established in cancer development and growth, then VEGF promotes angiogenesis and proliferation in the early stage of colorectal cancer development, and subsequently MCT4 promotes tumor cell growth through VEGF (Ullah et al., 2006). MCT4 inhibition can down-regulate VEGF expression in colorectal cancer cell lines, thereby inhibiting angiogenesis (Kim et al., 2018).

## MCT and programmed cell death

### MCT and ferroptosis

It is well established that PUFAs and MUFAs are the two major lipids that affect ferroptosis susceptibility in cancer cells. The ferroptosis process is driven by the PUFAs, of which the biosynthesis is catalyzed by the acyl-CoA synthetase long-chain family member 4 (ACSL4) (Das, 2019). It has also been reported that the SCD1-catalyzed MUFAs may replace PUFAs in the lipid membrane and reduce the accumulation of lipid ROS (Tesfay et al., 2019). The relative changes in the SCD1 and ACSL4 levels after MCT1-mediated lactate uptake suggest that the lactate-induced shifting in MUFAs and PUFAs production may act in concert to synergistically enhance the ferroptosis resistance in tumor cells by activating the AMPK-SREBP1-SCD1 pathway (Zhao et al., 2020). It has been reported that MCT4 can promote cell cycle progression and increase cell survival by altering cell cycle regulation and cell death mechanisms, especially late apoptosis/necrosis. At the same time, ferroptosis occurred when MCT4 was overexpressed, but not when MCT4 was expressedat baseline or not (Reuss et al., 2021).

### MCT and autophagy

Autophagy plays a complex dual role in the pathogenesis of tumors, which can act as an inhibitory factor or a promoting factor in the process of tumor development (White, 2015). Key proteins involved in autophagy, such as Beclin1, UVRAG, BIF- 1, and ATG, maintain cellular environment homeostasis in normal cells, prevent malignant transformation, and inhibit tumor initiation (Kriel and Loos, 2019). On the contrary, autophagy can also act as a tumor promoter by providing nutrients and energy to maintain the growth of cancer cells under hypoxic and hypotrophic conditions when tumors are formed, usually in the late stage of tumorigenesis (Schaaf et al., 2019; Mulcahy Levy and Thorburn, 2020).

Previous studies have shown that MCT1-mediated lactate uptake can activate the AMPK-SREBP1-SCD1 pathway and play a role in lipid metabolism and iron death (Zhao et al., 2020). MUFA has been reported to increase the fluidity and curvature of lipid bilayers, promote the formation of endoplasmic reticulum autophagosomes and activate autophagy. In turn, autophagy can also clear excess saturated fatty acids and damaged components, thereby counteracting cellular lipotoxicity, which is particularly important for the survival of tumor cells, especially during tumor initiation characterized by increased autophagy and up-regulation of SCD1 (Ascenzi et al., 2021). In hepatocellular carcinoma, inhibition of SCD1 can regulate autophagy by stimulating AMPK signaling (Huang et al., 2015). AMPK is an important energy signal sensor, which can phosphorylate ULK1 to form PI3K complex, promote glycolysis and induce autophagy to maintain ATP level (Chung et al., 2017; Yang et al., 2018). In the treatment of colorectal cancer (CRC), osimertinib (OSI) can up-regulate the protein level of MCT1 and subsequently induce autophagy in CRC cells through LKB1-mediated activation of AMPK, thereby antagonizing the anti-tumor effect of OSI, but the mechanism may be independent of the monocarboxylate transport function of MCT1 (Jin et al., 2019). The role of MCT1-mediated AMPK-SREBP1-SCD1 pathway in lipid metabolism and the induction of autophagy by its key molecules indicate the coupling of MCT1 in lipid metabolism and autophagy. In addition, autophagy can inhibit the phosphorylation and degradation of β-catenin by activating Wnt signaling, and the increased β-catenin is transported to the nucleus by transporters, thereby promoting the metastasis of hepatocellular carcinoma and glycolysis by increasing the transcription of MCT1 (Doherty et al., 2014).

It has also been suggested that inhibition of MCT4 can induce autophagy, thereby enhancing the cytotoxicity of NK cells and the ability to kill tumor cells (Long et al., 2018). Therefore, autophagy plays different roles according to the development stage and tissue type of cancer, which are opposite to some extent. These roles need to be fully elucidated when trying to formulate targeted therapy strategies for MCT and autophagy (Mulcahy Levy and Thorburn, 2020).

## MCTs and targeted drugs

MCTs, mainly MCT1 and MCT4, are overexpressed in solid tumors, and their mediated lactate shuttling between tumor cells plays an important role in maintaining the energy and PH balance necessary for tumor cell survival. Inhibition of these lactate transporters proved to be a novel anticancer strategy (Payen et al., 2020).

At present, several MCT inhibitors have been developed, but none of them are specific to MCT subtypes. Instead, a variety of molecular targets exist (Perez-Escuredo et al., 2016a). Early MCTs inhibitors, such as phloderin, quercetin, α-cyano4-hydroxycinnamate (CHC), and 4, 4' -diisocyanate- 2, 2′-disulfonic acid (DIDS), usually have a low affinity, poor specificity, and off-target effects (Wang et al., 2021). Recently, four novel selective MCT1 inhibitors have been reported with clinical potentials, such as AR-C155858, AZD3965, BAY-8002, and 7ACC2. AZD3965 is a small-molecule drug developed by Astrazeneca and is currently in phase I clinical trials. Although AZD3965, a derivative of AR-C155858, can inhibit both MCT1 and MCT2, the inhibitory effect on MCT1 was 6 times than MCT2 (Critchlow et al., 2012). The inhibitory effect of BAY-8002 on MCT1 is 5 times that of MCT2, and there is no off-target effect on MCT4 (Quanz et al., 2018).7ACC2 inhibits lactate inflow, but does not inhibit lactate outflow from cancer cells expressing MCT1/4 (Draoui et al., 2014). This may be related to the different conformational changes of MCT1 when these inhibitors bind to MCT1. It has been reported that MCT1 exhibits an outward-open conformation when combined with BAY-8002 and AZD3965, whereas in the presence of 7ACC2, it exhibits an inward-open conformation (Wang et al., 2021) (Table 3).

**TABLE 3:** Efficacy of MCTs inhibitors.

Inhibitor	Inhibition index	MCT1	MCT2	MCT4	Other targets	Ref
Syrosingopine	IC50(um)	∼ 2.5	ND	∼ 0.04	vesicular monoamine	Benjamin et al. (2018)
CHC	IC50(um)	150	ND	≥150	mitochondrial pyruvate carrier	Jonnalagadda et al. (2019)
compounds 1–9	IC50(um)	0.008–0.048	ND	0.011–0.085	—	Jonnalagadda et al. (2019)
phloretin	Ki(um)	14	5	41 ± 8.8 (4)	glucose transporters	Manning Fox et al. (2000)
DIDS	Ki(um)	434	ND	NI	bicarbonate transporters	Manning Fox et al. (2000)
quercetin	IC50(um)	14 ± 5	5 ± 2	ND	ERβ	Bröer et al. (1999)
ARC155858	Ki(nm)	2.3	＜10	NI	—	Ovens et al. (2010b)
AZD3965	Ki(nm)	1.6	9.6	NI	—	Critchlow et al. (2012)
BAY-8002	IC_50_ (nm)	1	5	>500	—	Quanz et al. (2018)
7ACC2	IC_50_ (nm)	11	ND	ND	—	Draoui et al. (2014)

Abbreviations:CHC: α-cyano-4-hydroxycinnamate; compounds 1–9: 2-methoxy-4-N,N-dialkyl cyanocinnamic acids 1–9; DIDS: 4,4′-diisothiocyanostilbene-2, 2′-disulphonate; ND, not determined; NI, no inhibition.

### Effects of MCT inhibitor

The only effective small-molecule selective MCT inhibitors developed to date are for MCT1, while very few for MCT4. Some small molecules, such as AZ93 and acridine flavine, have been reported in the literature, but their role has not been determined (Marchiq and Pouyssegur, 2016; Voss et al., 2017).

When MCT1 blocks the uptake of lactate with tumor cells, oxidative tumor cells can adapt by substrate switching. In other words, they take in more glucose from neighboring vascular regions and switch from lactate-driven OXPHOS to aerobic glycolysis for survival. However, glycolytic tumor cells that rely on metabolic symbiosis cannot perform substrate switch because of glucose deprivation, leading to apoptosis (Sonveaux et al., 2008). Therefore, inhibition of MCT1 overexpression in oxidized tumor cells can indirectly kill glycolytic tumor cells in hypoxic areas, where they are resistant to conventional chemotherapy, and it is the cause of tumor recurrence (Boasquevisque et al., 2018). This provides a new idea for cancer treatment. Once the tumor cells in the hypoxic zone are eliminated, more reactive tumor cells in the normoxic zone are more easily and effectively killed by conventional chemotherapy or radiotherapy.

MCT1 inhibition can also block the uptake of lactate by oxidized cancer cells to stromal cells, impairing the promotion of cancer cell proliferation by CAFs and the angiogenesis induced by lactate on endothelial cells (Vegran et al., 2011). In addition, MCT1 blockade usually results in the termination of lactate intake and accumulation of lactate in the tumor microenvironment. Increased extracellular lactate concentration can lead to reduced pyruvate and/or lactate release from glycolytic cells, intracytoplasmic acidification, and inhibition of glycolysis (Perez-Escuredo et al., 2016b).

Knockdown of MCT1 or blockade of lactate also promoted ferroptosis, but this effect was mainly achieved by regulating intracellular lipid metabolism rather than traditional regulators of ferroptosis, such as fibroblast specific protein 1 (FSP1) and glutathione peroxidase 4 (GPX4), which was not significantly altered in response to MCT1 inhibition (Zhao et al., 2020).

Although no effective small-molecule inhibitor of MCT4 has been reported, it is attractive considering the role of MCT4 in gravity flow metabolism. Inhibition of MCT4 can lead to the accumulation of lactate and H+ and cytoplasmic acidification in glycolytic tumor cells, and ultimately significantly increase cell death (Todenhofer et al., 2018). Secondly, inhibition of MCTs, especially MCT4, can reduce the production of a large number of antioxidants in the hypoxic zone, such as glutathione or lactate, and prevent tumor cells from entering the G0 quiescent phase by affecting the AMPK pathway and the mammalian target of rapamycin (mTOR) pathway, thereby enhancing the effect of radiation therapy (Brandstetter et al., 2021).

### Challenges of MCT1 inhibitors

AZD3965 is a potential selective MCT1 inhibitor. Its main mechanism of action is the specific inhibition of MCT1, which leads to the accumulation of intracellular lactic acid and reduction of intracellular PH, and feedback inhibition of glycolysis, thereby inhibiting the proliferation of tumor cells (Doherty et al., 2014). One of the challenges of AZD3965 in clinical application is its toxicity to heart and eye tissues, which can increase cardiac troponin levels and change retinal current patterns (Halford et al., 2017). Some studies have loaded AZD3965 into ultra-ph-sensitive nanoparticles to form nanopharmacology (AZD-UPS NP) (Huang et al., 2021). It remains stable at PH 7.4, but rapidly decomposes and releases AZD3965 when exposed to acidic PH, thus effectively inhibiting tumor growth. On the other hand, nanomedicine can significantly reduce the dose of oral AZD3965 and can decrease the accumulation and toxicity of the drug in the heart and liver tissues.

Another challenge with MCT1 inhibitors is that they are ineffective when MCT4 is overexpressed. This is a particularly serious defect because hypoxia leads to MCT4 expression in most tumors. In addition, MCT2 and MCT4 can overcompensate for the loss of MCT1 activity (Benjamin et al., 2018). It has been also reported that MCT4 is a “resistance factor” to MCT1 inhibitors (Polanski et al., 2014). Therefore, inhibition of both MCT1 and MCT4 is desirable.

It has been found that MCT1 inhibitor, at high concentrations of AR-C155858, does not cause cell death, but only reduces cell proliferation, even though MCT1 is the only lactate transporter (Benjamin et al., 2018). These results suggest that inhibition of lactate transport *per se* is not cytotoxic and that other mechanism may be involved in inducing cell lethality. Mitochondrial complex I (an NADH dehydrogenase) and lactate dehydrogenase (LDH) are the major cellular sources of NAD + regeneration required for glycolysis. In glycolytic tumor cells, LDH reduces pyruvate to lactate and simultaneously generates NAD+, which is beneficial to replenish the NAD + consumed by ATP generation in the glycolytic pathway. Inhibition of MCTs leads to intracellular lactic acid accumulation and feedback inhibition of LDH, thereby losing the ability of NAD + regeneration. In addition, metformin, an inhibitor of mitochondrial NADH dehydrogenase, can inhibit mitochondrial complex I, decrease NAD+/NADH ratio, and block glycolysis, followed by ATP production disorders and cell death (Benjamin et al., 2018). Therefore, pharmacological inhibition of MCT1 and MCT4 in combination with metformin is a potential tumor therapy.

Although AR-C155858 and AZD3965 can effectively inhibit lactate transport, their effects on cancer cell migration and invasion are very limited and cannot reduce tumor progression (Kong et al., 2016; Payen et al., 2017). Especially in HGF/C-Met metastatic tumors, such as breast and prostate cancer cells, pharmacological inhibition alone does not reduce the phosphorylation of liver growth factor (HGF) receptor c-Met and NF-κB activity and reduce the migration of cancer cells without MCT1 knockdown. Therefore, targeting MCT1 expression as well as transporter activity may be more effective than targeting transporter activity alone in anti-tumor metastasis (Gray et al., 2016).

## Conclusion

MCTs are upregulated in most types of human tumor cells, related to tumor stage and independent prognostic markers (Payen et al., 2020). Substrates of MCT include pyruvate, l-lactate, ketone bodies aceto-acetate and d- β-hydroxybutyrate, and short-chain fatty acid propionate and butyrate, in which lactate transportplays an important role in tumor metabolism (Wang et al., 2022). MCT1/MCT4-mediated lactate shuttling closely links tumor cells to each other, tumor cells to stromal cells, and tumor cells to immune cells. In the hypoxic and rapidly proliferating tumor cells, glycolysis and lactic acid fermentation are preferentially performed to obtain energy and maintain the growth of tumor cells (Liberti and Locasale, 2016). The lactate produced by the high glycolytic activity of tumor cells is excreted out of the cells by MCT4 to avoid intracellular acidification and death. Lactate transported by MCT1 and GPR81 is oxidatively phosphorylated in cells and affects lipid and amino acid metabolism to maximize the utilization of substrates, though oxidized tumor cells can use substrates such as glucose, lactate, lipid, and glutamine for metabolism (Pérez-Escuredo et al., 2016). In addition, the signal transduction of lactate can promote angiogenesis, immune escape, multidrug resistance, migration, and metastasis, and affect ferroptosis and autophagy, so as to avoid tumor cell death.

MCTs, especially MCT1 and MCT4, are potential targets for anti-tumor treatment. However, anti- MCTs drugs still face many problems, such as whether combined inhibition or highly selective inhibition of MCT is needed, and how to prevent tumor metastasis and reduce the toxic effects of drugs. Further research is needed to develop MCT-targeted drugs.
